# Excess mortality in radiotherapy-treated pituitary adenoma patients: 50-year follow-up

**DOI:** 10.1210/jendso/bvag115

**Published:** 2026-05-14

**Authors:** Anna Fitzgerald, James de Boisanger, Rosie Cooke, Daphne Traish, Anthony Swerdlow, Nicola Rosenfelder, Francesca Solda, Michael Brada

**Affiliations:** UCD School of Medicine, University College Dublin, Dublin D04 W6W8, Ireland; Neuro-oncology Unit, The Royal Marsden Hospital NHS Foundation Trust, London SW3 6JJ, UK; The Institute of Cancer Research, London SW7 3RP, UK; The Institute of Cancer Research, London SW7 3RP, UK; The Institute of Cancer Research, London SW7 3RP, UK; The Institute of Cancer Research, London SW7 3RP, UK; Neuro-oncology Unit, The Royal Marsden Hospital NHS Foundation Trust, London SW3 6JJ, UK; The Institute of Cancer Research, London SW7 3RP, UK; Neuro-oncology Unit, The Royal Marsden Hospital NHS Foundation Trust, London SW3 6JJ, UK; The Institute of Cancer Research, London SW7 3RP, UK; The Institute of Cancer Research, London SW7 3RP, UK; Department of Molecular and Clinical Cancer Medicine, University of Liverpool, Liverpool L69 7BE, UK

**Keywords:** pituitary adenoma, radiotherapy, cerebrovascular accidents, late toxicity

## Abstract

**Context:**

We report the long-term survival and the causes of excess mortality in a cohort of pituitary adenoma (PA) patients treated with radiotherapy, with or without prior surgery at up to 50 years of follow-up.

**Objective:**

To evaluate long-term survival and causes of excess mortality in patients with PAs treated with radiotherapy, with or without prior surgery, and to examine the relationship between treatment factors, particularly radiotherapy dose and mortality outcomes over extended follow-up.

**Methods:**

426 patients treated with radiotherapy at the Royal Marsden Hospital during 1962 and 1994 with or without prior surgery were followed up via the NHS Central Register to identify deaths and emigrations. Standardized mortality ratios (SMRs) and absolute excess risks (AERs) were calculated by comparing observed deaths with expected rates based on national age, sex, year, and period-specific mortality rates for England and Wales.

**Results:**

260 deaths were recorded compared to 144 expected (SMR 1.8; AER 140.7). The main contributors to excess mortality were cerebrovascular disease (SMR 4.9; AER 61.4), endocrine/metabolic disease (SMR 5.6), and mental and behavioral disorders (SMR 5.0). Both all-cause and cerebrovascular-specific mortality were associated with increasing radiotherapy dose and with the use of surgery.

**Conclusion:**

Patients with PAs treated with surgery and radiotherapy have an increased mortality compared to the general population, with the principal contributor cerebrovascular accident mortality. The observed dose-response suggests a potential relationship between radiotherapy and vascular morbidity. The findings highlight the need for long-term surveillance and proactive management of vascular risk factors and in patients requiring radiotherapy and avoiding excessive radiotherapy doses. Whether modern high-precision fractionated radiotherapy techniques reduce the risk of cerebrovascular morbidity and mortality requires long-term studies.

Pituitary adenomas (PAs)/pituitary neuroendocrine tumors, a clonal proliferation of anterior pituitary hormone-producing cells, account for 10% to 15% of all intracranial tumors [1]. The prevalence of PA is rising, likely due, at least in part, to increased detection through magnetic resonance (MR) imaging [2, 3]. Though benign in nature, they account for substantial morbidity and increased mortality [2].

The management of PAs is heterogenous and depends on tumor subtype and clinical presentation. While surgery is the primary therapy for many patients with secreting PA (SPA) and nonsecreting PAs (NSPA), a substantial proportion of patients, particularly those with prolactinomas, are primarily managed with medical therapy. Radiotherapy is typically reserved for secondary or tertiary settings, including progressive residual or recurrent disease, or for SPAs not controlled with surgery and medical treatment [4-6]. Radiotherapy has evolved over the decades, moving away from adjuvant radiotherapy following a subtotal resection. Similarly, the delivery techniques of fractionated radiotherapy have improved, with increased conformality of treatment and greater avoidance of surrounding normal structures, although similar dose/fractionation schedules of known effectiveness continue to be used [4, 5].

Several studies have investigated survival in PA patients, highlighting the impact of hormonal imbalance and cerebrovascular accidents (CVAs) on outcomes [7-13]. Large cohort studies of patients with both SPAs and NSPAs treated with conventionally fractionated radiotherapy reported excellent local tumor control/progression-free survival (PFS) with >90% PFS at 10 years [5, 6, 14, 15] and 88% at 20 years [5, 16]. Many studies also suggested a risk of long-term complications and associated excess mortality [5, 6, 17-20]. These include hypopituitarism, CVAs, and second brain tumors, although, in the absence of randomized studies, the exact causes and the likely contribution of radiotherapy to the presumed late toxicities are difficult to define [21, 22].

We present up to 50-year follow-up data from an expanded cohort of patients treated in England reported previously [17], compare the mortality with that expected contemporaneous national mortality rates for England and Wales, and assess the potential contribution of factors including CVA, endocrine, nutritional, and metabolic diseases, and cancer.

## Patients and methods

A cohort of 426 patients, resident in the United Kingdom and treated with radiotherapy at the Royal Marsden Hospital during 1962 to 1994 (expanded from a previously report cohort [17]) was retrospectively reviewed. The patient, disease, and treatment characteristics are shown in Table 1. Prior to referral for radiotherapy, 347 patients underwent surgery. All patients received radiotherapy using a 3-field technique, with a majority treated to a dose of 45 Gy in 25 fractions (range 40-55 Gy in 20-30 fractions) (Table 1).

**Table 1:** Descriptive characteristics of the cohort

Characteristic	No. of patients	Person-years of follow-up
Sex	247	4686
Male	179	3589
Female		
Year of radiotherapy		
1962-1969	100	2461
1970-1979	141	2790
1980-1989	128	2200
1990-1994	57	824
Age at start of radiotherapy (years)		
15-19	11	312
20-29	37	1135
30-39	72	1984
40-49	88	1924
50-59	112	1930
60-69	76	766
70-79	30	223
Type of tumor		
Nonsecreting	263	4862
Secreting	139	2919
Acromegaly/raised GH	74	1443
Prolactinoma	49	1153
ACTH-secreting	12	262
Other	4	61
Unknown	24	494
Type of surgery		
None	79	1797
Trans-cranial	224	4444
Trans-sphenoidal	120	1995
Other	3	38
Radiotherapy dose (Gy)		
≤44	116	2723
45-49	212	3835
50-55	98	1717
Total	426	8275

All patients were followed up via the National Health Service Central Register to identify deaths and emigrations. Emigrations were censored on the date of departure. The register was last accessed on the 5th May 2011, and all patients still receiving follow-up were censored at this date. This amounts to 50 years from the date of the first patient in the study receiving radiotherapy (1962) and 17 years since the last treatment of the last patient included. Patients were also censored at the date of death and on reaching age 85, to avoid problems of miscertification of cause of death in old age. Of the 426 eligible patients, 14 were lost to follow-up, 3 were registered as having emigrated, 27 were censored at age 85, and 122 were recorded as alive as of the 5th May 2011. A total of 8275 person-years of follow-up were accumulated in the cohort, and 260 deaths were identified during follow-up. Causes of death were taken as the underlying causes from the death certificates, and all deaths were coded to the 9th revision of International Classification of Diseases (ICD9).

Mortality was assessed by computing standardized mortality ratios (SMRs) as the ratio of the number of deaths observed in our study to the number of deaths expected based on national age, sex, year, and period-specific mortality rates for England and Wales. Absolute excess risk (AER) presents this ratio in terms of the difference in absolute risk per 10 000 population.

## Results

### Mortality and causes of death

In total, 260 deaths were observed compared to 144 expected at national rates, giving an SMR of 1.8 (95% CI 1.6-2.0) and an AER of 140.7 (95% CI 103.7-181.3) in patients with PA (Table 2). There were excess deaths from cerebrovascular disease (SMR 4.9 (95% CI 3.8-6.2), AER 61.4 (95% CI 43.6-82.8)), excess deaths from endocrine, nutritional and metabolic disease (SMR 5.6 (95% CI 3.1-10.1), AER 10.9 (95% CI 4.3-21.4)), and excess deaths from mental and behavioral disorders (SMR 5.0 (95% CI 2.2-11.1), AER 5.8 (95% CI 1.2-14.3). There were no excess deaths observed for malignancy overall (SMR 0.9 (95% CI 0.6-1.2), AER −7.4 (95% CI −20.7-9.5)).

**Table 2:** Risk of death by selected causes in patients treated for pituitary adenoma

ICD9 code	Cause of death	Obs	Exp	SMR (95%CI)	AER (95% CI)
0010-1398	Infectious and parasitic disease	2	0.74	2.7 (0.7, 10.8)	1.5 (−0.6, 7.8)
1400-2089	All malignancies	37	43.15	0.9 (0.6, 1.2)	−7.4 (−20.7, 9.5)
1620-1629	Lung cancer	8	12.15	0.7 (0.3, 1.3)	−5.0 (−10.5, 4.4)
1740-1759	Breast cancer	6	2.94	2.0 (0.9, 4.5)	3.7 (−0.9, 12.2)
2400-2799	Endocrine, nutritional, and metabolic disease	11	1.97	5.6 (3.1, 10.1)	10.9 (4.3, 21.4)
2500-2509	Diabetes	4	1.59	2.5 (0.9, 6.7)	2.9 (−0.6, 10.5)
2900-3199	Mental and behavioral disorders	6	1.21	5.0 (2.2, 11.1)	5.8 (1.2, 14.3)
3200-3899	Nervous system disease	9	2.20	4.1 (2.1, 7.9)	8.2 (2.3, 18.0)
3900-4599	Circulatory disease	129	64.55	2.0 (1.7, 2.4)	77.9 (52.1, 107.2)
4100-4109	Acute myocardial infarction	22	24.30	0.9 (0.6, 1.4)	−2.8 (−12.7, 10.9)
4100-4149	Ischemic heart disease	49	39.67	1.2 (0.9, 1.6)	11.3 (−4.1, 30.3)
4160-4169; 4200-4299	Other heart disease	9	4.11	2.2 (1.1, 4.2)	5.9 (0.0, 15.7)
4300-4379	Cerebrovascular disease	64	13.18	4.9 (3.8, 6.2)	61.4 (43.6, 82.8)
4300-4309	Subarachnoid hemorrhage	4	0.87	4.6 (1.7, 12.3)	3.8 (0.3, 11.3)
4600-5199	Respiratory disease	29	17.68	1.6 (1.1, 2.4)	13.7 (2.1, 29.0)
4800-4839; 4850-4869	Pneumonia	18	7.68	2.3 (1.5, 3.7)	12.5 (3.6, 25.1)
5200-5799	Digestive disease	7	4.66	1.5 (0.7, 3.2)	2.8 (−2.2, 11.8)
5800-6299	Genito-urinary disease	2	1.55	1.3 (0.3, 5.2)	0.5 (−1.6, 6.9)
7100-7399	Musculoskeletal system and connective tissue disease	2	0.69	2.9 (0.7, 11.7)	1.6 (−0.5, 7.9)
8000-9999	Accident and violence	2	3.48	0.6 (0.1, 2.3)	−1.8 (−3.9, 4.5)
0000-9999	All causes	260	143.55	1.8 (1.6, 2.0)	140.7 (103.7, 181.3)

Abbreviations: AER, absolute excess risk per 10 000; CI, confidence interval; SMR, standardized mortality ratio.

Both the all-cause SMR and AER were higher for females (SMR 2.2 (95% CI 1.8-2.6); AER 154.0 (95% CI 101.5-214.8)) than males (SMR 1.6 (95% CI 1.4-1.9) and AER 130.6 (95% CI 80.1-187.5)) (Table 3). Based on age at start of radiotherapy, AER increased with each 10-year increase in age (*P*_trend_ < .001), but the trend for SMR was not statistically significant (*P*_trend_ = .11). There was no significant difference in SMR and AER in relation to the time since start of radiotherapy (Table 3).

**Table 3:** Risk of death from all causes in relation to sex, age at start of radiotherapy, and time since start of radiotherapy

	Obs	Exp	SMR (95% CI)	AER (95% CI)
Sex				
Male	158	96.80	1.6 (1.4, 1.9)	130.6 (80.1, 187.5)
Female	102	46.74	2.2 (1.8, 2.6)	154.0 (101.5, 214.8)
Age at start of radiotherapy (years)				
15-19	3	0.41	7.3 (2.3, 22.6)	82.9 (6.6, 267.5)
20-29	6	3.96	1.5 (0.7, 3.4)	18.0 (−15.5, 80.2)
30-39	30	14.17	2.1 (1.5, 3.0)	79.8 (30.6, 144.4)
40-49	53	28.89	1.8 (1.4, 2.4)	125.3 (56.2, 210.1)
50-59	87	47.82	1.8 (1.5, 2.2)	203.0 (113.3, 308.2)
60-69	61	31.37	1.9 (1.5, 2.5)	386.7 (199.5, 613.1)
70-79	20	16.93	1.2 (0.8, 1.8)	138.0 (−211.7, 627.3)
			*P_trend_* = *.11*	*P_trend_* *<* *.001*
Time since start of radiotherapy (years)				
0-4	53	24.81	2.1 (1.6, 2.8)	142.8 (75.4, 225.5)
5-9	50	25.75	1.9 (1.5, 2.6)	142.8 (66.9, 236.6)
10-14	30	24.94	1.2 (0.8, 1.7)	34.8 (−32.2, 122.9)
15-19	42	23.99	1.8 (1.3, 2.4)	151.6 (52.9, 276.0)
20-24	33	17.94	1.8 (1.3, 2.6)	178.2 (56.5, 336.1)
25-29	27	11.50	2.3 (1.6, 3.4)	287.9 (116.9, 516.0)
30-34	11	7.81	1.4 (0.8, 2.5)	96.4 (−70.1, 358.8)
≥35	14	6.80	2.1 (1.2, 3.5)	293.2 (34.8, 679.6)
			*P_trend_* = *.86*	*P_trend_* = *.16*

Abbreviations: AER, absolute excess risk per 10 000; CI, confidence interval; SMR, standardized mortality ratio.

### Cerebrovascular deaths

There were 64 cerebrovascular deaths with an SMR of 4.9 (95% CI 3.8-6.2) and an AER of 61.4 (95% CI 43.6-82.8) (Table 4). Both SMR and AER were higher for females (6.1 (95% CI 4.4-8.6) and 77.0 (95% CI 48.3-114.2)) than males (4.0 (95% CI 2.8-5.6) and 49.5 (95% CI 28.3-77.2)). AER increased with increasing age at the time of radiotherapy (*P*_trend_ < .001). The trend for increasing SMR with age at radiotherapy did not reach statistical significance (*P*_trend_ = .11).

**Table 4:** Risk of death from cerebrovascular disease in relation to sex, age at start of radiotherapy, and time since start of radiotherapy

	Obs	Exp	SMR (95% CI)	AER (95% CI)
Sex				
Male	31	7.80	4.0 (2.8, 5.6)	49.5 (28.3, 77.2)
Female	33	5.38	6.1 (4.4, 8.6)	77.0 (48.3, 114.2)
Age at start of radiotherapy (years)				
15-19	0	0.02	0.0 (., .)	−0.6 (., 117.5)
20-29	1	0.20	5.1 (0.7, 36.1)	7.1 (−1.5, 47.4)
30-39	9	0.91	9.9 (5.2, 19.1)	40.8 (16.2, 81.5)
40-49	9	2.13	4.2 (2.2, 8.1)	35.7 (10.3, 77.7)
50-59	21	4.26	4.9 (3.2, 7.6)	86.7 (45.3, 144.2)
60-69	17	3.34	5.1 (3.2, 8.2)	178.3 (85.7, 311.6)
70-79	7	2.34	3.0 (1.4, 6.3)	209.6 (21.5, 543.1)
			*P_trend_* = *.11*	*P_trend_* *<* *.001*
Time since start of radiotherapy (years)				
0-4	10	2.38	4.2 (2.3, 7.8)	38.6 (12.2, 81.1)
5-9	12	2.54	4.7 (2.7, 8.3)	55.7 (21.6, 108.5)
10-14	11	2.29	4.8 (2.7, 8.7)	59.8 (22.0, 119.4)
15-19	12	2.17	5.5 (3.1, 9.8)	82.8 (34.0, 158.2)
20-24	4	1.60	2.5 (0.9, 6.7)	28.4 (−6.1, 102.3)
25-29	7	0.96	7.3 (3.5, 15.2)	112.1 (34.4, 250.0)
30-34	3	0.67	4.5 (1.4, 13.9)	70.5 (−1.5, 244.8)
≥35	5	0.57	8.7 (3.6, 21.0)	180.3 (42.8, 451.8)
			*P_trend_* = *.36*	*P_trend_* = *.33*

Abbreviations: AER, absolute excess risk per 10 000; CI, confidence interval; SMR, standardized mortality ratio.

### Impact of PA type and radiotherapy dose

There was no significant difference in all-cause mortality between SPA and NSPA (SMR 1.8 (95% CI 1.6-2.1) vs 2.0 (95% CI 1.6-2.5), respectively, and AER 160.7 (95% CI 109.5-218.0) vs 119.4 (95% CI 66.1-183.0), respectively) (Table 5). Both SMR and AER increased with increasing radiotherapy dose (*P*_trend_ < .001 and .04, respectively). There was no significant difference in CVA mortality between SPAs and NSPAs (SMR 4.6 (95% CI 3.4-6.2) vs 6.1 (95% CI 3.8-9.6)) respectively; AER 67.7 (95% CI 43.5-98.1) vs 51.5 (95% CI 26.4-87.3) respectively (Table 5). The SMR for CVA mortality increased with increasing dose of radiotherapy, with SMR ranging from 2.9 for doses <44 Gy to 8.4 for doses 50 to 55 Gy (*P*_trend_ = .002). The difference in AER did not reach statistical significance (*P*_trend_ = .17). The SMR for CVA mortality was 2.2 (95% CI 1-4.5) in patients treated with radiotherapy alone, compared with 6.0 (95% CI 4-4.8) and 5.1 (95% CI 3-8.6) for patients treated surgically using a trans-cranial and trans-sphenoidal approach, respectively.

**Table 5:** Risk of death from all causes and cerebrovascular disease in relation to type of tumor and radiotherapy dose

	Obs	Exp	SMR (95% CI)	AER (95% CI)
**All causes**				
Type of tumor				
Nonsecreting	174	95.85	1.8 (1.6, 2.1)	160.7 (109.5, 218.0)
Secreting	71	36.14	2.0 (1.6, 2.5)	119.4 (66.1, 183.0)
Unknown	15	11.55	1.3 (0.8, 2.2)	69.9 (−63.8, 267.1)
Radiotherapy dose (Gy)				
≤44	75	51.95	1.4 (1.2, 1.8)	84.6 (25.9, 154.5)
45-49	124	68.51	1.8 (1.5, 2.2)	144.7 (90.3 206.9)
50-55	61	23.08	2.6 (2.1, 3.4)	220.9 (137.3, 322.0)
			*P*_*trend*_ *<* *.001*	*P*_*trend*_ = *.04*
**Cerebrovascular disease**				
Type of tumor				
Nonsecreting	42	9.10	4.6 (3.4, 6.2)	67.7 (43.5, 98.1)
Secreting	18	2.97	6.1 (3.8, 9.6)	51.5 (26.4, 87.3)
Unknown	4	1.11	3.6 (1.3, 9.6)	58.4 (−0.5, 184.8)
Radiotherapy dose (Gy)				
≤44	15	5.20	2.9 (1.7, 4.8)	36.0 (11.7, 71.7)
45-49	34	6.19	5.5 (3.9, 7.7)	72.5 (45.3, 107.7)
50-55	15	1.79	8.4 (5.1, 13.9)	77.0 (38.5, 133.7)
			*P_trend_* = *.002*	*P_trend_* = *.17*

Abbreviations: AER, absolute excess risk per 10 000; CI, confidence interval; SMR, standardized mortality ratio.

## Discussion

We report long-term outcomes, up to five decades after treatment, in patients with PAs and assess the mortality in comparison to the general population. We previously reported a cohort of 334 patients treated between 1962 and 1986 at 30 years follow-up [17]. In the previous cohort of 334 patients, the overall SMR was 1.7 with cerebrovascular disease identified as a major contributor to excess mortality [17]. The current expanded cohort demonstrates a similar but slightly higher SMR of 1.8, with consistent findings regarding cerebrovascular mortality. CVA remained the principal cause of the excess mortality, representing 25% (64/260) of deaths. The SMRs for endocrine causes and CVA were 5.6 and 4.9, respectively. There were no excess deaths observed for malignancy overall (SMR 0.9).

Other published studies suggested a risk of long-term complications in PA cohorts treated with radiotherapy, such as hypopituitarism, CVAs, and second brain tumors [5, 6, 17-20]. A retrospective study of 100 patients treated with surgery and radiotherapy for PAs between 1983 and 2003 reported 3 CVAs with radiological evidence of an infarct within or near the irradiated areas [19]. Development of second, presumed radiation-induced tumors has been suggested as a late complication following radiotherapy for PAs [5, 18, 20, 23]. We previously reported a relative risk (RR) of developing a second brain tumor of 10.5 and a cumulative risk at 20 years of 2.4% [20].

Two large population-based studies have subsequently provided more robust estimates of this risk. A cohort study of approximately 9000 patients with hypopituitarism (majority with PAs) reported a significantly increased risk of secondary intracranial tumors following pituitary radiotherapy, particularly meningiomas and malignant brain tumors [24]. More recently, a multicenter study of approximately 3700 pituitary tumor patients with long-term imaging follow-up found an increased RR of secondary brain tumors in irradiated patients compared with nonirradiated controls, although the AER remained low [25]. These findings support an association between pituitary radiotherapy and secondary brain tumors, while emphasizing that the absolute risk is small and must be balanced against the benefits of tumor control.

In the absence of randomized studies, the contribution of radiotherapy to the presumed late-radiation toxicities is difficult to disentangle from other potential causes [21, 22]. A systematic review evaluating the radiotherapy-related risk of ischemic stroke in PA patients concluded that no significant difference in the risk of ischemic stroke in irradiated vs nonirradiated patients could be identified [21]. Similarly, a national surveillance study of 806 patients in the Netherlands reported that the incidence of second intracranial tumors did not differ between irradiated and nonirradiated patients [22]. However, the presence of a dose–response relationship between radiotherapy dose and late effects in our data suggests a potential causal link. However, it is likely that higher radiation doses were reserved for larger tumors, and the potential increased risk of CVA mortality due to tumor size cannot be excluded.

Several studies investigated mortality and survival in PA patients generally and have highlighted the impact of hormonal imbalance and CVAs [7-13]. A prospective study of a cohort of patients with hypopituitarism assessed the association between premature mortality and hypopituitarism, and the reported excess mortality was attributed to cardiovascular, respiratory, and cerebrovascular causes [13]. A nationwide study of 31 898 patients examined the incidence and mortality of PA patients in Korea. Mortality was attributed to cardiovascular disease (stroke and myocardial infarction) and tumor-related factors such as hypopituitarism and endocrine dysfunction [7]. However, the study did not provide an analysis of treatments received, and the absence of such data limits the ability to assess a relationship between radiotherapy and stroke incidence. Other studies highlight a relationship between hormonal deficiencies and increased mortality in patients with NSPAs [9-12], especially in women with hypopituitarism [9, 11]. Our results suggest higher mortality in females compared to males (SMR 2.2 vs 1.6, respectively).

Interestingly, all-cause mortality and cerebrovascular mortality were similar between patients with NSPAs and SPAs, which was an unexpected finding. One potential explanation is that nonsecreting tumors may have been larger at presentation and therefore more frequently required transcranial/trans frontal surgical approaches, which are associated with higher perioperative morbidity and a greater risk of hypopituitarism. In contrast, a substantial proportion (57%) of the secreting tumors in this cohort were prolactinomas, a subtype not typically associated with increased cerebrovascular or cardiovascular comorbidity [26]. This difference in tumor biology and treatment characteristics may have attenuated expected differences in long-term mortality between NSPAs and SPAs.

The key finding in this study (as previously [17]) is the excess of cerebrovascular deaths (SMR 4.9) as the principal determinant of increased mortality. The etiology of CVAs in this patient cohort is not known and likely multifactorial including endocrine, surgical, and radiation-related risk factors. The observed higher cerebrovascular mortality in females compared to males may reflect, in part, the loss of vasculoprotective effects of estrogen, particularly in the context of. Hypopituitarism or treatment-induced gonadal dysfunction. Estrogen deficiency has been associated with adverse vascular profiles and increased risk of cerebrovascular disease, which may contribute to the sex differences observed in this cohort [27, 28].

The excess mortality observed from mental and behavioral disorders represents an important and relatively unexplored finding. This may reflect an increased burden of depression, neurocognitive impairment, or self-harm in this patient population. Comparable findings have been reported in patients with other benign intracranial tumors treated with radiotherapy, such as meningiomas, where long-term neuropsychological sequelae and reduced quality of life have been described [29, 30]. These findings suggest that neuropsychiatric outcomes may contribute meaningfully to long-term morbidity and mortality.

Patients with endocrine dysfunction (acromegaly, Cushing Disease (CD), and hypopituitarism) are at a raised risk of CVA and mortality [7, 31, 32]. Hypopituitarism is a recognized late complication of both surgery and radiotherapy, with incidence increasing with higher radiation doses, and is associated with increased cardiovascular and cerebrovascular mortality [13, 18, 31]. In acromegaly, the risk may be related to the effect of excess GH secretions associated with vascular disease, cardiomyopathy, diabetes, and hypertension [5, 21, 32, 33]. The enhanced risk in CD is also likely at least in part the direct consequence of cortisol hypersecretion on the cardiovascular system [31, 34]. Hypopituitarism is also associated with a raised risk of vascular disease with potential impact on the risk of CVA [31, 35].

In our previous study, a higher risk of CVA mortality was noted in patients who had surgery than those who did not (RR = 5.19 vs RR = 1.33; *P* = .02) [17], and this remains a finding in the present study (SMR >5). While the reason for the association between surgery and increased risk of CVA is not clear, latent surgical damage possibly potentiated by radiotherapy has been proposed as a contributing factor [17]. Importantly, surgery in this context may act as a marker of tumor size, extent, invasiveness, or perioperative morbidity rather than a direct causal factor, and this should be considered when interpreting the observed associations. One of the most common complications following pituitary surgery is hypopituitarism [35, 36]. The rates of postoperative hypopituitarism range from 10% to 25% dependent on tumor size, degree of invasion, and neurosurgeon competency [35] and may be a contributing factor [17, 31, 35], although the majority of patients have regular endocrine assessment and timely hormone replacement therapy.

Radiotherapy for PAs has been associated with increased incidence of CVA and CVA-specific mortality [17, 37, 38], although not all studies support this [21]. In this study, all-cause mortality and CVA-specific mortality increased with radiotherapy dose, which suggests a potential relationship (Table 5), although it is not possible to exclude tumor size as a contributing factor as it is likely that larger radiotherapy dose was reserved for patients with larger tumors. While tumor size cannot be excluded as a contributing factor, it is also possible that patients receiving higher radiation doses had a greater baseline cerebrovascular risk. Unmeasured confounders such as hypertension, smoking, obesity, hyperlipidemia, genetic predisposition, and socioeconomic factors may have been more prevalent in patients presenting with more advanced disease and are well-established contributors to cerebrovascular disease [39, 40]. The absence of detailed data on these variables, as well as on hypopituitarism status and its management, limits the ability to adjust for confounding. Therefore, these findings should be interpreted as hypothesis-generating rather than establishing a causal relationship between radiotherapy and increased mortality.

The relationship between cranial radiotherapy and CVAs has been studied in other brain pathologies and populations. A PENTEC meta-analysis to determine the risk of cerebral vasculopathy after cranial irradiation in the pediatric population also reported a higher risk of stroke with higher radiation dose. Patients who received 54 Gy and reached 45 years of age had a risk of stroke of 6.7% to 12%, compared to a baseline risk of 0.5% to 1% [41]. A retrospective case–control study of 116 patients treated with radiotherapy for primary brain tumors between 1975 and 2006, and 85 controls (nonirradiated patients) with at least 10 years of follow-up reported a stroke prevalence of 36% and 8% in the radiotherapy and nonradiotherapy cohorts, respectively [42]. The largest study, of 19 565 patients from the SEER database who received radiotherapy for primary brain tumors (with a median follow-up of 12.75 years), reported a higher 10-year stroke-specific mortality in patients receiving radiotherapy to tumors near central arterial structures, compared to those with tumors distant from the central vasculature [43].

The use of stereotactic techniques, by providing greater conformality and smaller planning target margins, aims to reduce the radiation dose to neighboring structures. Although the dose to the carotid artery and central vasculature may be reduced, the clinical impact on posttreatment CVA and overall mortality is unclear. Future analysis of outcomes following different radiation techniques may help gain a better understanding of the effect of radiation particularly on the cerebral vasculature and the impact on the risk of CVA and mortality in patients with PA.

Our study has several strengths, most notably its exceptionally long duration of follow-up, which to our knowledge is the longest reported in this patient group. The large cohort size and high completeness of follow-up data further enhance the reliability and relevance of the observed associations. However, the study has limitations. As this study examines mortality rather than event incidence, the findings cannot be extrapolated to nonfatal outcomes, and no conclusions can be drawn regarding the risk of nonfatal cerebrovascular events such as transient ischemic attacks or nonfatal strokes. Cause of death was obtained from death certificates, which may be subject to misclassification; nonetheless, this limitation applies equally to population comparisons and therefore does not bias RR estimates. Cause of death assignment based solely on death certificates may be less reliable in patients with pituitary disease. In a large hypopituitarism cohort using multiple complementary data sources, several deaths classified as cardiovascular or cerebrovascular on death certificates were reclassified as endocrine-related causes such as adrenal crisis, indicating potential overestimation of vascular mortality when death certificates are used alone [44]. In addition, censoring follow-up at age 85 was chosen to reduce potential misclassification of cause-specific mortality at advanced age; however, this approach excludes a clinically relevant period during which cerebrovascular events are common. While all-cause mortality estimates are unlikely to be materially affected by this censoring, future analyses without age-based censoring would be valuable to further assess the robustness of cause-specific findings. Lastly, radiotherapy exposures in this cohort largely reflect older treatment techniques, which may not fully represent outcomes associated with more modern approaches. Despite these considerations, our findings contribute important insights into the late vascular effects of PA treatment.

## Conclusion

PAs, although benign, are associated with a number of long-term health risks, particularly cerebrovascular mortality. The disease and its metabolic consequences, as well as radiotherapy and other treatment modalities, such as surgery, may contribute to the increased mortality in this patient population. We report an excess in all-cause and cerebrovascular mortality compared to the normal population. Cerebrovascular disease was the leading contributor to excess deaths with a dose–response relationship observed between radiotherapy dose and CVA mortality and increased risk with the use of surgery. While in the absence of randomized data, causality cannot be established. The results underscore the need for careful long-term follow-up, attention to vascular risk factors, and consideration of radiotherapy dose in patients requiring radiotherapy. Long-term studies in PA populations receiving more localized radiotherapy techniques are needed to assess if the current methods of treatment delivery are associated with a lower risk of cerebrovascular disease.

## Data Availability

Some or all datasets generated during and/or analyzed during the current study are not publicly available but are available from the corresponding author on reasonable request.

## References

[1] Lake MG, Krook LS, Cruz SV. Pituitary adenomas: an overview. Am Fam Physician. 2013;88(5):319‐327. 24010395

[2] Faltermeier CM, Magill ST, Blevins LS, Aghi MK. Molecular biology of pituitary adenomas. Neurosurg Clin N Am. 2019;30(4):391‐400. 10.1016/j.nec.2019.05.001 31471046

[3] Aflorei ED, Korbonits M. Epidemiology and etiopathogenesis of pituitary adenomas. J Neurooncol. 2014;117(3):379‐394. 10.1007/s11060-013-1354-5 24481996

[4] Rosenfelder N, Solda F, Brada M. Radiotherapy in pituitary tumours. In: DeGroot's Endocrinology: Basic Science and Clinical Practice. 8th ed. Elsevier; 2023:1‐14.

[5] Feingold KR, Anawalt B, Blackman MR, et al Endotext. MDText.com, Inc.; 2000.

[6] Kim MY, Kim JH, Oh YK, Kim E. Long-term outcomes of surgery and radiotherapy for secreting and non-secreting pituitary adenoma. Radiat Oncol J. 2016;34(2):121‐127. 10.3857/roj.2016.01683 PMC4938346 27306775

[7] Oh JS, Kim HJ, Hann HJ, et al Incidence, mortality, and cardiovascular diseases in pituitary adenoma in Korea: a nationwide population-based study. Pituitary. 2021;24(1):38‐47. 10.1007/s11102-020-01084-6 32949324

[8] Chen C, Hu Y, Lyu L, et al Incidence, demographics, and survival of patients with primary pituitary tumors: a SEER database study in 2004-2016. Sci Rep. 2021;11(1):15155. 10.1038/s41598-021-94658-8 PMC8313564 34312470

[9] Lindholm J, Nielsen EH, Bjerre P, et al Hypopituitarism and mortality in pituitary adenoma. Clin Endocrinol (Oxf). 2006;65(1):51‐58. 10.1111/j.1365-2265.2006.02545.x 16817819

[10] Olsson DS, Bryngelsson IL, Ragnarsson O. Time trends of mortality in patients with non-functioning pituitary adenoma: a Swedish nationwide study. Pituitary. 2017;20(2):218‐224. 10.1007/s11102-016-0764-8 PMC5357499 27743172

[11] Olsson DS, Nilsson AG, Bryngelsson IL, Trimpou P, Johannsson G, Andersson E. Excess mortality in women and young adults with nonfunctioning pituitary adenoma: a Swedish nationwide study. J Clin Endocrinol Metab. 2015;100(7):2651‐2658. 10.1210/jc.2015-1475 25946030

[12] O'Reilly MW, Reulen RC, Gupta S, et al ACTH and gonadotropin deficiencies predict mortality in patients treated for nonfunctioning pituitary adenoma: long-term follow-up of 519 patients in two large European centres. Clin Endocrinol (Oxf). 2016;85(5):748‐756. 10.1111/cen.13141 PMC5082557 27327840

[13] Tomlinson JW, Holden N, Hills RK, et al Association between premature mortality and hypopituitarism. West Midlands prospective hypopituitary study group. Lancet. 2001;357(9254):425‐431. 10.1016/s0140-6736(00)04006-x 11273062

[14] McCollough WM, Marcus RB, Rhoton AL, Ballinger WE, Million RR. Long-term follow-up of radiotherapy for pituitary adenoma: the absence of late recurrence after greater than or equal to 4500 cGy. Int J Radiat Oncol Biol Phys. 1991;21(3):607‐614. 10.1016/0360-3016(91)90677-v 1907958

[15] Sasaki R, Murakami M, Okamoto Y, et al The efficacy of conventional radiation therapy in the management of pituitary adenoma. Int J Radiat Oncol Biol Phys. 2000;47(5):1337‐1345. 10.1016/s0360-3016(00)00503-4 10889388

[16] Brada M, Rajan B, Traish D, et al The long-term efficacy of conservative surgery and radiotherapy in the control of pituitary adenomas. Clin Endocrinol (Oxf). 1993;38(6):571‐578. 10.1111/j.1365-2265.1993.tb02137.x 8334743

[17] Brada M, Ashley S, Ford D, Traish D, Burchell L, Rajan B. Cerebrovascular mortality in patients with pituitary adenoma. Clin Endocrinol (Oxf). 2002;57(6):713‐717. 10.1046/j.1365-2265.2002.01570.x 12460319

[18] Sherlock M, Ayuk J, Tomlinson JW, et al Mortality in patients with pituitary disease. Endocr Rev. 2010;31(3):301‐342. 10.1210/er.2009-0033 20086217

[19] Snead FE, Amdur RJ, Morris CG, Mendenhall WM. Long-term outcomes of radiotherapy for pituitary adenomas. Int J Radiat Oncol Biol Phys. 2008;71(4):994‐998. 10.1016/j.ijrobp.2007.11.057 18403133

[20] Minniti G, Traish D, Ashley S, Gonsalves A, Brada M. Risk of second brain tumor after conservative surgery and radiotherapy for pituitary adenoma: update after an additional 10 years. J Clin Endocrinol Metab. 2005;90(2):800‐804. 10.1210/jc.2004-1152 15562021

[21] van Westrhenen A, Muskens IS, Verhoeff JJC, Smith TRS, Broekman MLD. Ischemic stroke after radiation therapy for pituitary adenomas: a systematic review. J Neurooncol. 2017;135(1):1‐11. 10.1007/s11060-017-2530-9 PMC5658475 28660317

[22] van Varsseveld NC, van Bunderen CC, Ubachs DH, et al Cerebrovascular events, secondary intracranial tumors, and mortality after radiotherapy for nonfunctioning pituitary adenomas: a subanalysis from the Dutch National Registry of growth hormone treatment in adults. J Clin Endocrinol Metab. 2015;100(3):1104‐1112. 10.1210/jc.2014-3697 25574793

[23] Yamanaka R, Abe E, Sato T, Hayano A, Takashima Y. Secondary intracranial tumors following radiotherapy for pituitary adenomas: a systematic review. Cancers (Basel). 2017;9(8):103. 10.3390/cancers9080103 PMC5575606 28786923

[24] Burman P, van Beek AP, Biller BM, Camacho-Hübner C, Mattsson AF. Radiotherapy, especially at young age, increases the risk for de novo brain tumors in patients treated for pituitary/sellar lesions. J Clin Endocrinol Metab. 2017;102(3):1051‐1058. 10.1210/jc.2016-3402 28359095

[25] Hamblin R, Vardon A, Akpalu J, et al Risk of second brain tumour after radiotherapy for pituitary adenoma or craniopharyngioma: a retrospective, multicentre, cohort study of 3679 patients with long-term imaging surveillance. Lancet Diabetes Endocrinol. 2022;10(8):581‐588. 10.1016/S2213-8587(22)00160-7 35780804

[26] Toma ID, Niculescu DA, Găloiu SA, Trifănescu RA, Poiană C. Comparative mortality in pituitary adenomas subtypes: a tertiary referral center study. Endocrine. 2025;87(2):782‐787. 10.1007/s12020-024-04073-y PMC11811481 39425840

[27] Mendelsohn ME, Karas RH. The protective effects of estrogen on the cardiovascular system. N Engl J Med. 1999;340(23):1801‐1811. 10.1056/NEJM199906103402306 10362825

[28] Rosano GM, Panina G. Oestrogens and the heart. Therapie. 1999;54(3):381‐385. 10500455

[29] Dijkstra M, van Nieuwenhuizen D, Stalpers LJ, et al Late neurocognitive sequelae in patients with WHO grade I meningioma. J Neurol Neurosurg Psychiatry. 2009;80(8):910‐915. 10.1136/jnnp.2007.138925 18653549

[30] Waagemans ML, van Nieuwenhuizen D, Dijkstra M, et al Long-term impact of cognitive deficits and epilepsy on quality of life in patients with low-grade meningiomas. Neurosurgery. 2011;69(1):72‐78; discussion 78-9. 10.1227/NEU.0b013e318212badb 21346660

[31] Osorio RC, Oh JY, Choudhary N, Lad M, Savastano L, Aghi MK. Pituitary adenomas and cerebrovascular disease: a review on pathophysiology, prevalence, and treatment. Front Endocrinol (Lausanne). 2022;13:1064216. 10.3389/fendo.2022.1064216 PMC9791098 36578965

[32] Esposito D, Ragnarsson O, Granfeldt D, Marlow T, Johannsson G, Olsson DS. Decreasing mortality and changes in treatment patterns in patients with acromegaly from a nationwide study. Eur J Endocrinol. 2018;178(5):459‐469. 10.1530/EJE-18-0015 29483205

[33] Melmed S, Kaiser UB, Lopes MB, et al Clinical biology of the pituitary adenoma. Endocr Rev. 2022;43(6):1003‐1037. 10.1210/endrev/bnac010 PMC9695123 35395078

[34] Ferraù F, Korbonits M. Metabolic comorbidities in Cushing's syndrome. Eur J Endocrinol. 2015;173(4):M133‐M157. 10.1530/EJE-15-0354 26060052

[35] Kim SY . Diagnosis and treatment of hypopituitarism. Endocrinol Metab (Seoul). 2015;30(4):443‐455. 10.3803/EnM.2015.30.4.443 PMC4722397 26790380

[36] Prete A, Corsello SM, Salvatori R. Current best practice in the management of patients after pituitary surgery. Ther Adv Endocrinol Metab. 2017;8(3):33‐48. 10.1177/2042018816687240 PMC5363454 28377801

[37] Brada M, Burchell L, Ashley S, Traish D. The incidence of cerebrovascular accidents in patients with pituitary adenoma. Int J Radiat Oncol Biol Phys. 1999;45(3):693‐698. 10.1016/s0360-3016(99)00159-5 10524424

[38] Erridge SC, Conkey DS, Stockton D, et al Radiotherapy for pituitary adenomas: long-term efficacy and toxicity. Radiother Oncol. 2009;93(3):597‐601. 10.1016/j.radonc.2009.09.011 19900729

[39] O'Donnell MJ, Chin SL, Rangarajan S, et al Global and regional effects of potentially modifiable risk factors associated with acute stroke in 32 countries (INTERSTROKE): a case-control study. Lancet. 2016;388(10046):761‐775. 10.1016/S0140-6736(16)30506-2 27431356

[40] Yusuf S, Hawken S, Ounpuu S, et al Obesity and the risk of myocardial infarction in 27,000 participants from 52 countries: a case-control study. Lancet. 2005;366(9497):1640‐1649. 10.1016/S0140-6736(05)67663-5 16271645

[41] Waxer JF, Wong K, Modiri A, et al Risk of cerebrovascular events among childhood and adolescent patients receiving cranial radiation therapy: a PENTEC normal tissue outcomes comprehensive review. Int J Radiat Oncol Biol Phys. 2024;119(2):417‐430. 10.1016/j.ijrobp.2022.06.079 PMC12123681 36057476

[42] Ibáñez-Juliá MJ, Picca A, Leclercq D, et al Late-onset vascular complications of radiotherapy for primary brain tumors: a case-control and cross-sectional analysis. J Cancer Surviv. 2024;18(1):59‐67. 10.1007/s11764-023-01350-z PMC10867030 37142871

[43] Aizer AA, Du R, Wen PY, Arvold ND. Radiotherapy and death from cerebrovascular disease in patients with primary brain tumors. J Neurooncol. 2015;124(2):291‐297. 10.1007/s11060-015-1839-5 26080799

[44] Burman P, Mattsson AF, Johannsson G, et al Deaths among adult patients with hypopituitarism: hypocortisolism during acute stress, and de novo malignant brain tumors contribute to an increased mortality. J Clin Endocrinol Metab. 2013;98(4):1466‐1475. 10.1210/jc.2012-4059 23457412

